# Complete remission in a patient with metastatic gastric cancer receiving tislelizumab combined with chemotherapy: a case report

**DOI:** 10.3389/fonc.2023.1147636

**Published:** 2023-05-10

**Authors:** Zhe Zhu, Pei-Lin Dai, Shuai Han, Enming Qiu, Yu Wang, Zhou Li

**Affiliations:** ^1^ The Second School of Clinical Medicine, Southern Medical University, Guangzhou, China; ^2^ Department of General Surgery, Zhujiang Hospital, Southern Medical University, Guangzhou, China; ^3^ Department of Pathology, Zhujiang Hospital, Southern Medical University, Guangzhou, China

**Keywords:** immune checkpoint inhibitor, tislelizumab, advanced gastric cancer, complete remission, case report

## Abstract

The prognosis for patients with advanced gastric cancer (AGC) is poor, with limited treatment options available due to the difficulty of resection. In recent years, chemotherapy and immunotherapy for AGC have shown promising efficacy. However, there is a controversy regarding the surgery of primary tumors and/or metastases in patients with stage IV gastric cancer after systematic therapy. Here, we present a 63-year-old retired female of AGC with supraclavicular metastasis with positive PD-L1 and tumor mutational burden-high (TMB-H). After receiving 8 cycles of capecitabine and oxaliplatin (XELOX) in combination with tislelizumab, the patient achieved complete remission (CR). No evidence of recurrence was identified during follow-up. To the best of our knowledge, this is the first case of AGC with supraclavicular metastasis who achieved CR after treatment with tislelizumab. The mechanism of CR was discussed by genomic and recent clinical studies. The results indicated that programmed death ligand-1 (PD-L1) combined positive score (CPS) ≥5 may serve as a clinical indication and standard for chemo-immune combination therapy. In combination with other similar reports, patients with microsatellite instability-high/defective mismatch repair (MSI-H/dMMR), (TMB-H), and positive PD-L1 had better sensitivity to tislelizumab. The patient recovered successfully except for symptoms of gastrointestinal hemorrhage during treatment, which may be associated with the treatment cycle and age. Immunotherapy with tislelizumab has been well-established in the treatment of malignant melanoma, lung cancer, and clear-cell kidney cancer, but its efficacy and safety for esophageal and gastric cancers remain to be validated. The CR of our patient suggested the prospects of tislelizumab in the immunotherapy of gastric cancer. Additionally, a watch-and-wait (WW) method maybe offered for patients with AGC who achieved complete clinical remission (CCR) after immune combination therapy if the patient was older or in poor physical condition.

## Introduction

As one of the most common malignant tumors in China, gastric cancer (GC) has the third highest incidence and mortality rate among all malignant tumors, with the second highest incidence and third highest mortality rate among malignant tumors in males (1). The 5-year survival rate after surgery for early-stage GC is as high as 95%. However, the early symptoms of GC are not obvious and difficult to detect and are often diagnosed only when the disease deteriorates and progresses to an advanced stage (2). In this setting, patients with metastatic advanced gastric cancer (AGC) have a poor prognosis and extremely limited treatment. The 5-year survival rate for AGC has been reported to be only 8.8%-14.9% (3, 4), and complete remission (CR) is rare.

Currently, the primary goal of treatment in AGC is to alleviate symptoms and prolong survival. Chemotherapy serves as the primary treatment, and numerous studies and meta-analyses have revealed that systemic chemotherapy is superior to best supportive care (BSC) in treating AGC. Compared to BSC, chemotherapy prolongs the overall survival (OS) of patients by almost 6.7 months (5). From the 1970s to the 1980s, scientists conducted the exploration of antibody therapy and cytokine therapy. In the last decade, the discovery of immune checkpoint proteins (cytotoxic T lymphocyte-associated antigen-4 [CTLA-4] and programmed death 1 [PD-1]) represents a breakthrough in the field of tumor therapy (6–8), especially the discovery of PD-1/programmed death ligand-1 (PD-L1) signaling pathway and the emergence of inhibitors targeting PD-1/PD-L1 pathway are changing the current strategy of tumor therapy. In recent years, PD-1 inhibitors have also shown promising efficacy in AGC. However, PD-1 monotherapy with imperfect endpoints has suffered setbacks and faced major challenges in several phase III clinical trials (9, 10). As increasingly chemo-immune combination therapy is being explored in GC, the success of the CheckMate-649 trial has brought great confidence to first-line chemo-immune combination therapy. A total of 1581 patients were randomly assigned to nivolumab plus chemotherapy or chemotherapy alone to evaluate the efficacy of first-line nivolumab plus chemotherapy for AGC. The results showed that nivolumab plus chemotherapy significantly improved OS (13.8 vs. 11.6 months; hazard ratio [HR]=0.71, *p*<0.001) and progression-free survival (PFS) (7.7 vs. 6.9 months; [HR]=0.68; *p*<0.001) compared to chemotherapy alone. Subsequently, the data were disclosed from the ORIENT-16 study, which enrolled 650 patients randomly assigned to sintilimab plus chemotherapy or chemotherapy alone in a 1:1 ratio. At a median follow-up of 18.8 months, the median OS of patients who received sintilimab plus chemotherapy vs. chemotherapy alone was 15.2 months (95% confidence interval [CI], 12.9-18.4 months) vs. 12.3 months (95% CI, 11.3-13.8 months). Following the success of numerous clinical trials, PD-1 inhibitor plus chemotherapy has become the new standard first-line treatment option for AGC, and the chemo-immune combination therapy has been recommended by the 2022 National Comprehensive Cancer Network (NCCN) and 2022 Chinese Society of Clinical Oncology (CSCO) guidelines (9, 10). PD-1 inhibitors confer a remarkable survival benefit in patients with AGC, whereas immunotherapy only prolonged PFS and OS by 1 month and 2-3 months, respectively (9). In addition, surgical resection is inseparable from chemotherapy and immunotherapy in the treatment of gastric cancer. Several retrospective studies have evaluated the effect of surgery for primary tumors and/or metastatic lesions on patients with stage IV GC. The results suggest that surgery may be related to prolonged survival in some patients, such as those aged ≤70 years with one metastatic site, with one incurable site and a good response to preoperative systemic chemotherapy, or with liver metastasis that may be completely resected (11–13). However, the role of surgical intervention in metastatic GC remains an open question. Recent research has revealed that a multimodal approach including perioperative chemotherapy and/or conversion surgery may offer a better prognosis for selected patients and that the efficacy of surgery during immunochemotherapy varies depending on the biological characteristics of AGC (14).

It is still controversial whether to perform surgery after chemo-immune combination therapy, and there are no relevant systematic studies. The highlight of our case was that the patient was found to have the opportunity to undergo surgery at the end of the treatment and CR of the GC and metastases was achieved during the resection. The CR of our case is very rare compared to current clinical studies such as CheckMate-649 and ORIENT-16. We treated patient with capecitabine and oxaliplatin (XELOX) in combination with tislelizumab. Tislelizumab is an anti-programmed death receptor-1 (PD-1) inhibitor designed to help the body’s immune cells detect and fight tumors. Tislelizumab is specifically designed to minimize binding to the Fcγ receptor in macrophages. In the variable region of the antibody that binds to the PD-1 target, tislelizumab has a unique binding epitope that distinguishes it from nabulizumab and pablizumab, with a large overlap of the binding surface on PD-1 with PD-L1, enabling complete blockade of PD-1 binding to PD-L1 (15). The significant therapeutic effect may provide a reference for clinicians or provide a basis for subsequent relevant clinical trials for new treatment options.

## Case description

### Demographic information

On January 16, 2022, a 63-year-old retired female who presented with upper abdominal distension and pain for more than six months accompanied by weakness and progressive weight loss was admitted to our hospital. The patient complained of a history of hysterectomies. The patient had no other comorbidities, tobacco or alcohol dependence, or familial and hereditary disease. The physical examination showed epigastric tension and tenderness.

### Diagnostic assessments

**Figure 1:**
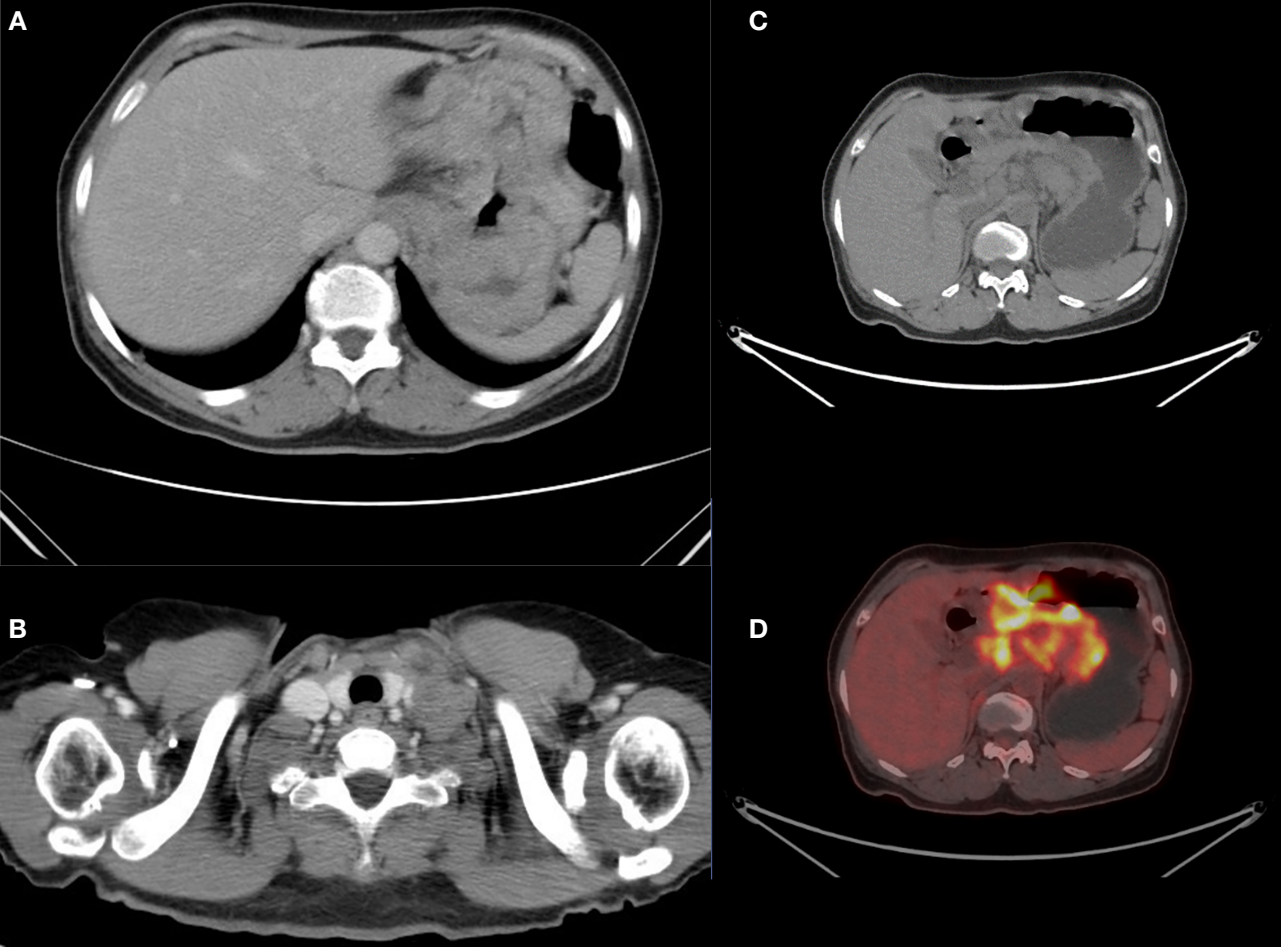
Computed tomography (CT) showed multiple enlarged lymphatic in the supraclavicular or infraclavicular fossa **(A)** and uneven enhancement and thickening in the gastric body fundus **(B)**. The positron emission tomography (PET)-CT scan **(C, D)** revealed all lesions were metabolically active.

The patient underwent gastroscopy in other hospitals and the pathology suggested gastric cancer. However, the patient received no medical treatment and transferred to our hospital for further examination. Contrast-enhanced computed tomography (CT) (**Figure 1A**) of the thorax and abdomen showed uneven enhancement and thickening and soft tissue mass in the gastric body fundus, with carcinoembryonic antigen (CEA) serum level of 3.5 ng/mL and glycoantigen (CA724) serum level of 11.4 KU/L. Multiple enlarged lymph nodes were observed in the lesser gastric curvature, retroperitoneum, and parietal aorta, partially fused and poorly demarcated from the adjacent gastric wall. Additionally, multiple enlarged lymphatic shadows observed in the supraclavicular or infraclavicular fossa were recognized as supraclavicular lymph node metastasis (**Figure 1B**), which was proved by subsequent pathological examination. Simultaneously, positron emission tomography (PET)-CT revealed uneven thickening and formation of masses in the gastric wall of the cardia-fundus-gastric body, and multiple enlarged lymph nodes on the side of lesser gastric curvature, retroperitoneum, parietal abdominal aorta, and left supraclavicular and infraclavicular fossa (**Figures 1C, D**), all of which were metabolically active. Gastroscopy revealed a pericircular mass around the gastric sinus, which involved the gastric horn, the lesser curvature of the gastric body, and the cardia, resulting in deformation and narrowing of the pyloric hilum. Pathological findings confirmed Epstein-Barr virus (EBV)-associated hypofractionated adenocarcinoma with the following immunohistochemical parameters: cytokeratin (CK) (+), CK7 (-), P40 (-), CEA (partially+), human epidermal growth factor receptor 2 (HER-2) (0+), Ki-67 (+, 30%). The patient showed microsatellite stable (MSS) phenotype (MLH1/MSH2/MSH6/PMS2). The next-generation sequencing (NGS) of circulating tumor DNA analysis (Burning Rock, Guangzhou, China) revealed a positive expression of PD-L1 (combined positive score [CPS]=30), a negative expression of HER-2 with an Eastern Cooperative Oncology Group (ECOG) performance status of 1, gene mutations of *PIK3CB* p.K57T (27.12%) and *PIK3CB* p.D1067V (24.12%), and no mutation in epidermal growth factor receptor (*EGFR*) and *TP53*. A tumor mutational burden value of 20.94 mutations per megabase (muts/Mb) was evaluated as tumor mutational burden-high (TMB-H), which may be a biomarker for immunotherapy benefit. Finally, the patient was diagnosed with pT4NxM1 stage IV GC. An 8-cycle of capecitabine plus oxaliplatin (XELOX) in combination with tislelizumab was determined based on family opinions, TMB, and PD-L1 CPS levels under the guidance of the multi-disciplinary team (MDT) pattern.

### Therapeutic interventions and follow-up

After the physician team explained the condition and discussed it with the patient, the patient herself and her family requested immunotherapy combined with chemotherapy. From February 13 to September 6, 2022, the patient was treated with intravenous capecitabine (1000 mg twice a day on days 1-14) plus oxaliplatin (130 mg/m^2^ once on day 1) (XELOX) in combination with tislelizumab (200 mg once on day 1) as first-line chemotherapy for eight cycles (3 weeks per cycle) due to her supraclavicular lymph node metastasis and her satisfactory ECOG PS score, PD-L1 expression, and TMB score. Subsequently, a contrast-enhanced CT scan demonstrated normalization of the previously unevenly thickened gastric wall (**Figure 1**). However, the treatment was suspended after 4 cycles as the patient presented with gastrointestinal bleeding, which was confirmed as bleeding gastric ulcer. The treatment was restarted after giving symptomatic treatment such as fasting, hemostasis, and nutritional support for one month. At the end of eight cycles of treatment, the decision to perform laparoscopic total gastrectomy and esophago-jejunostomy was determined under the guidance of the MDT pattern due to the strong desire of the patient who wanted to eliminate both primary and secondary lesions and achieved disease-free state.

**Figure 2:**
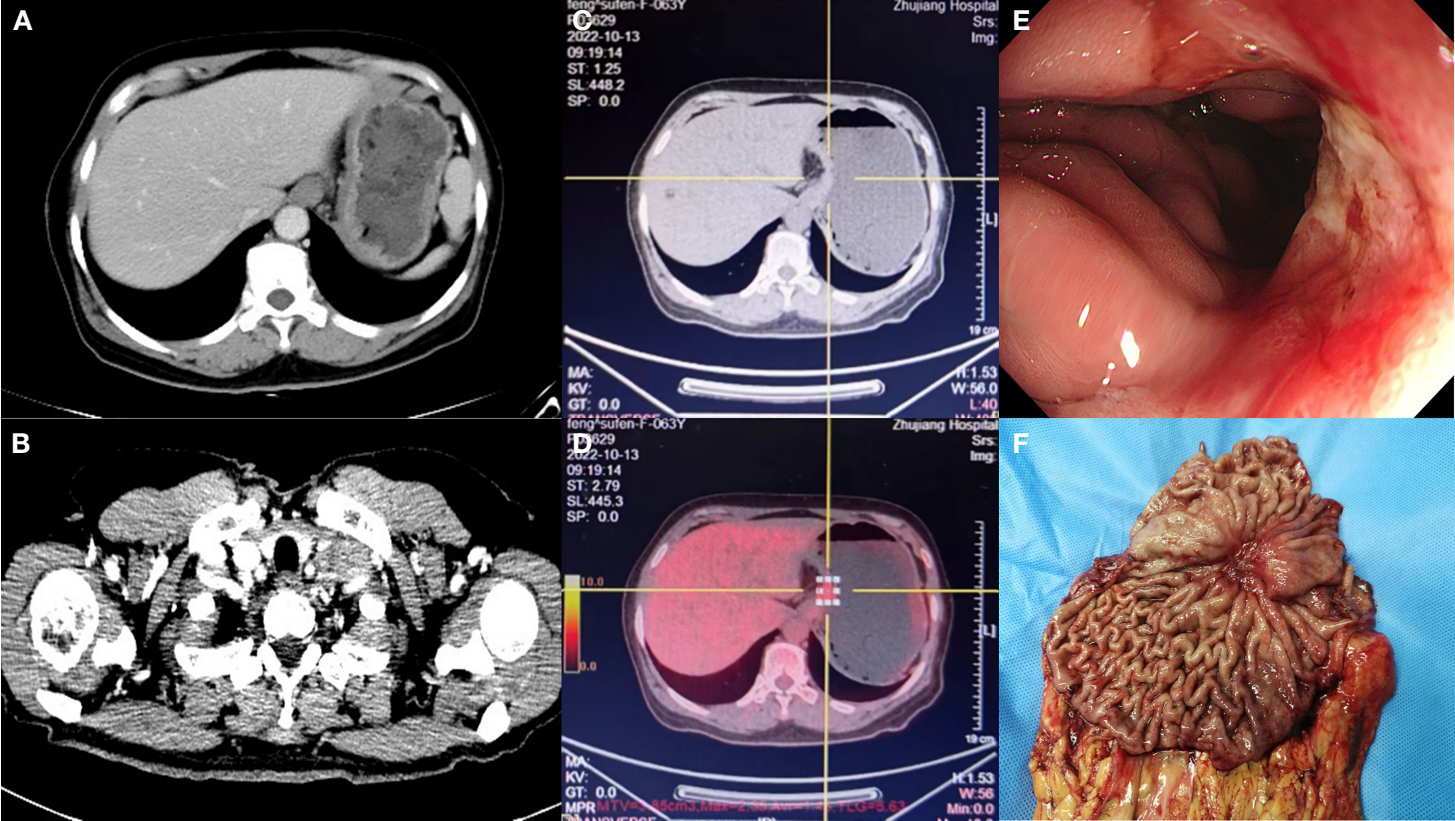
Preoperative contrast-enhanced computed tomography (CT) showed that the uneven thickening of the gastric wall and multiple enlarged lymphatic in the left cervical was reduced **(A, B)**. The positron emission tomography (PET)-CT showed that the previously uneven thickening of gastric wall and the previously metabolically active lymph nodes in the left clavicular region was reduced, and metabolism dropped to the background **(C, D)**. Gastroscopy after treatment revealed an ulcerated mass **(E)**. The primary focus of stomach **(F)**.

During the preoperative examination, dramatic results were observed. Preoperative contrast-enhanced CT of the thorax and abdomen (**Figures 2A, B**) showed that the uneven thickening of the gastric wall in the fundus and body of the stomach was reduced, and the multiple enlarged lymph nodes on the side of the lesser gastric curvature, retroperitoneum, parietal abdominal aorta, and left supraclavicular and infraclavicular fossa were reduced and shrunk. Similarly, compared to the PET/CT on February 08, 2022, preoperative PET/CT (**Figures 2C, D**) revealed a dramatic reduction in the previously uneven thickening of the gastric wall in the cardia-fundus-gastric body, a reduction in the size of the previously metabolically active lymph nodes in the left clavicular region, abdominal cavity, retroperitoneum, bilateral parietal iliac vessels, and anterior iliac, and a decline in metabolism to the background, which was considered as a post-treatment change. Gastroscopy revealed an ulcerated mass in the fundus and the lesser curvature of the stomach, with lesions involving the cardia, gastric horn, and part of the gastric sinus (**Figure 2E**). Subsequently, preoperative pathological examination of superficial mucosa (gastric body) suggested chronic active non-atrophic gastritis, which was consistent with ulcerative changes. No malignant lesions were observed, and the immunohistochemical showed creatine kinase (CK) (+).

**Figure 3:**
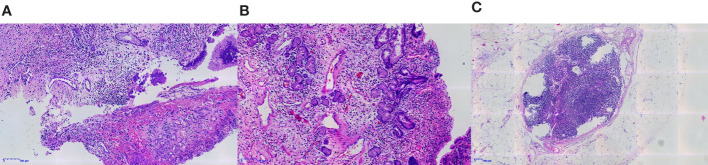
The gastric tissue sent for examination showed ulcerative changes, and no tumor cells remained **(A, B)**. No residual tumor cells were seen in the lymph nodes sent for examination **(C)**.

**Figure 4:**
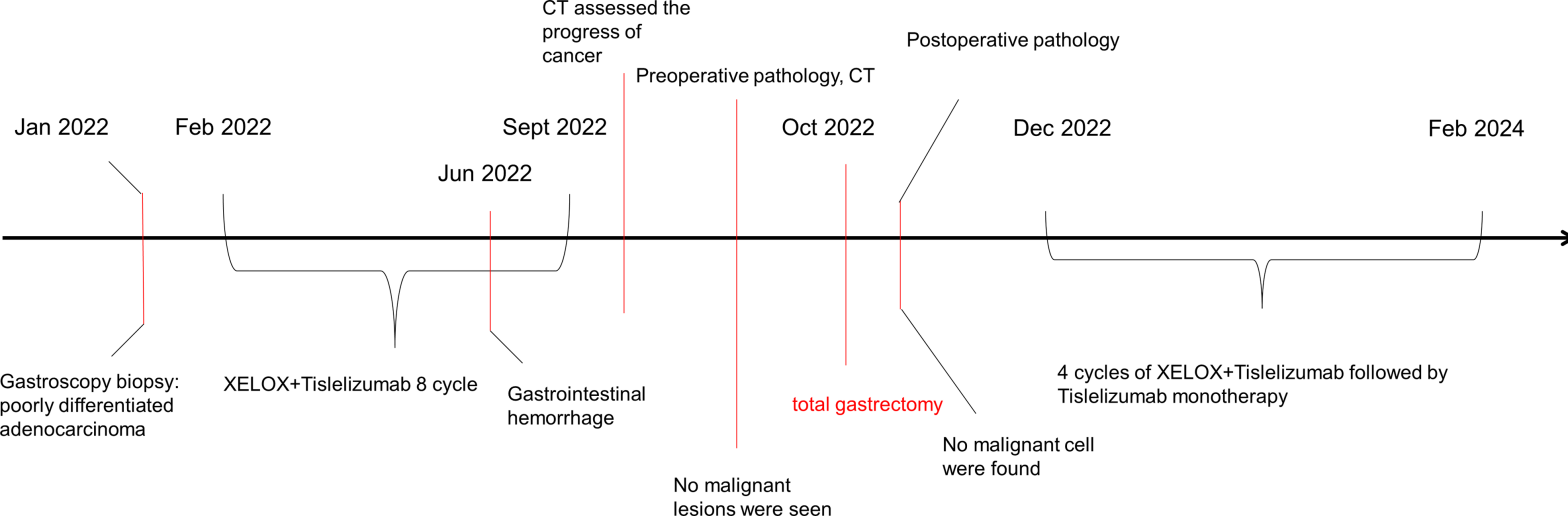
Clinical course over time. It includes the treatments, diagnostic procedures and timing of disease progression.

Thereafter, surgical intervention was implemented on October 14, 2022. The primary focus of the stomach is shown in **Figure 2F**. The pathological results showed (**Figures 3A–C**) : 1. the gastric tissue sent for examination showed ulcerative changes, no cancer cell residue was seen; Tumor Regression Score (NCCN standard) grade 0: No viable cancer cells, including lymph nodes; immunohistochemistry: 3#: CK (normal mucosal epithelium +); 5#: CK (normal mucosal epithelium +); 9#: CK (normal mucosal epithelium +); 2. no residual cancer cells were seen in the sent examination (proximal and distal margins) and in the self examination of both margins; 3. (Lymph node 2, lymph node 3, lymph node 4, lymph node 5) and self-examination of lymph nodes, no cancer cells were seen in lymph nodes (0/1, 0/3, 0/2, 0/1, 0/5, 0/1); among them (Mupa node 4, lymph node 5), necrosis and granuloma were seen, which were considered as post-treatment reaction; immunohistochemistry: 16#: CK (-); 4. (Lymph node 1) No lymphatic structures were seen, and no cancer cells were seen. The patient received a total of 8 cycles of XELOX, and the cumulative dose of oxaliplatin was 1040 mg/m^2^. No dose-related adverse events (AEs), such as peripheral neurotoxic symptoms, were observed. Simultaneously, the cumulative dose of tislelizumab was 1600 mg, and no AEs associated with tislelizumab (such as hypothyroidism, hyperthyroidism, and skin rash) were observed. The multidisciplinary discussion concluded that all examinations had confirmed that the primary and secondary lesions of the patient had been completely removed. The timeline is displayed in **Figure 4**. The patient was discharged on day 7 after surgery in good condition and resumed a normal life, demonstrating that CR was achieved with tislelizumab combined with XELOX chemotherapy. However, considering that the patient had stage IV GC, under the guidance of the multidisciplinary team (MDT), we still recommended that patients receive 4-5 cycles of chemotherapy combined Tislelizumab followed by tislelizumab monotherapy maintenance up to 2 years. Currently, the patient has received 3 cycles treatment in Dec 2022, Feb 2023 and Mar 2023 respectively, no evidence of recurrence was identified during follow-up.

## Discussion

PD-1/PD-L1 targeted immunotherapy represents a new era in tumor treatment, which may be the most effective and safest way to treat tumors by fully utilizing and mobilizing tumor-killing T cells. The KEYNOTE-012 (16) study, announced on June 2016, officially opened the path to immunotherapy in GC. In the KEYNOTE-012 study, patients (including the US, Japanese, Korean and Taiwanese populations) with PD-L1-positive AGC received pembrolizumab monotherapy (10 mg/kg) as a ≥third-line treatment. The objective remission rate (ORR), median PFS, median OS, and the incidence of grade 3-4 treatment-related AEs (TRAEs) was 22%, 1.9 months, 11.4 months, and 13%, respectively, and there were no treatment-related deaths. The KEYNOTE-012 study was the first to demonstrate the promising antitumor activity and manageable toxicities of pembrolizumab in AGC. In recent years, immune checkpoint inhibitors (ICIs), represented by natalizumab and pembrolizumab, have been investigated in several clinical studies for the treatment of AGC, and sufficient data are available to confirm their safety and efficacy (16–19).

Meanwhile, the efficacy of chemo-immune combination therapy for AGC has also been confirmed by several clinical trials. A randomized, multicenter, phase III clinical trial (ATTRACTION-4) showed that chemo-immune combination therapy further improved the PFS (10.45 vs. 8.34 months) of Asian patients compared to chemotherapy alone (20). Our case demonstrated that chemo-immune combination therapy significantly prolonged the median OS and PFS of the patients, respectively, which was consistent with the results of the latest CheckMate 649 trial (9). However, the clinical indications and adapted molecular and immunological characteristics for combination therapy remain obscure.

Currently, researchers are focusing on molecular markers, including microsatellite instability-high (MSI-H), PD-L1, EBV, TMB, and HER-2 (21), with a view to these markers accurately predicting prognosis in the future. There is evidence that the addition of ICI to chemotherapy lacks benefit in low PD-L1-expressing gastroesophageal adenocarcinoma (GEAC) tumors, while it is shown to be effective in those with high PD-L1 expression (22, 23). In the CheckMate 649 trial (9), first-line chemotherapy combined with anti-PD-1 achieved encouraging efficacy in treating patients with positive PD-L1 expression (CPS ≥5), with prolonged median OS and median PFS. The ATTRACTION-4 trial (20) investigated the efficacy of nivolumab plus oxaliplatin-based chemotherapy for patients with HER2-negative advanced gastric or gastroesophageal junction cancer, regardless of PD-L1 expression. However, the results showed that nivolumab combined with oxaliplatin-based chemotherapy only significantly improved PFS, but not OS. The discrepant outcomes suggested that PD-L1 CPS ≥5 may serve as a clinical indication and standard for chemo-immune combination therapy.

Through a series of reviews of the relevant literature, a similar case report was noted (24). A 69-year-old patient with AGC of MSI-H, TMB-H, EBV positive, PD-L1 positive, and HER-2 negative received a combination regimen of chemotherapy and tislelizumab. After 3 cycles of treatment, pathological examination confirmed that the lesions had disappeared, which was similar to our case in terms of gene expression and clinical efficacy. ICIs have been approved in the United States for tumors exhibiting defective mismatch repair (dMMR), MSI, or TMB-H (25). Despite a positive correlation between TMB-H and GC incidence has been reported in meta-analyses, evidence also suggests that patients with high PD-L1 expression and TMB-H derive favorable survival benefits from ICIs (26). Meanwhile, it was reported in a study that PD-L1 positivity, Interferon-gamma (IFN-γ)-related gene signature, and TMB-H were positively associated with the clinical benefit of tislelizumab in advanced GEAC (27). It is uncertain whether this is a coincidence, but it is reasonable to speculate that patients with MSI-H/dMMR, TMB-H, and positive PD-L1 expression have better sensitivity to tislelizumab. If this hypothesis is confirmed by subsequent relevant studies, the studies will be practice-changing and identify indications for immunotherapy. The limitation of the presented case is the absence of the EBV burden of both lesions, which has a high correlation with the incidence and prognosis of GC (28).

A related study and several case reports with the application of tislelizumab found that *TP53*-mutant tumor cells improved the sensitivity of patients to tislelizumab by promoting the upregulation of PD-L1 expression and T-cell infiltration (29–31). Contrary to the above findings, the NGS of our patient did not show *TP53* mutation but a high mutation of *PIK3CB*, and our patient might also benefit from PD-1/PD-L1 ICIs.

Notably, the patient presented with gastrointestinal bleeding during chemotherapy. The study (32) from Tian et al. indicated that GC patients with acute non-surgical related hemorrhage during chemotherapy had a poor prognosis. In contrast to this finding, our patient achieved a CR even despite the development of gastrointestinal bleeding caused by gastric ulcers. This anomaly is a question worthy of exploration, and the following reasons might partially explain it. On the one hand, gastrointestinal bleeding often leads to the interruption of anti-tumor therapy, and the fasting and anemia-related symptoms after bleeding reduce the opportunity to continue chemotherapy, leading to a decrease in patient survival. However, the treatment of gastrointestinal bleeding in our patient was prompt and did not interfere with the cycle of chemotherapy plus immunotherapy. On the other hand, a favorable prognosis is also associated with older age. Notably, in the context of GC combined with gastrointestinal bleeding, older patients have a better prognosis, which might be attributed to the increased susceptibility of the gastric mucosa to injury at older ages (33).

There are obstacles in the subsequent treatment of patients after chemo-immune combination therapy. One of the most important issues is whether the patients should undergo subsequent surgery, which is often related to whether the physical condition of patients can tolerate the surgery. For patients with AGC who achieve complete clinical remission (CCR), the watch-and-wait (WW) method rather than direct sequential surgical resection of the primary lesion may provide a new treatment option for a patient with an elderly or poor physical condition. However, it is still difficult to determine whether a patient has achieved CR by preoperative imaging and tumor biomarker assessment (34). The WW method can make a preliminary determination based on the tumor biomarkers of the patient. If multiple biomarkers against this treatment regimen are positive, it may suggest that the patient has a better prognosis for this approach. The WW strategy has been widely used in colon cancer (35). The excellent rectal preservation and pelvic tumor control of the WW strategy have been demonstrated, yet it may have worse survival in patients with local regrowth (36).

Recent studies have demonstrated both the short-term benefits of less blood loss, postoperative pain and faster recovery and the oncologic safety of laparoscopy (37). Thus, it is worth mentioning that we tried Laparoscopic total gastrectomy (LTG) on a patient even he had stage IV gastric cancer with metastasis before chemo-immune combination therapy. Currently, for early gastric cancer, Laparoscopic Distal gastrectomy(LDG) for stageIgastric cancer is strongly recommended as a standard treatment in guidelines (38). Additionally, clinical trial like JCOG1401 has also confirmed the feasibility of Laparoscopic total gastrectomy (LTG) and it is weakly recommended as a treatment for early gastric cancer (39). For advanced gastric cancer, although non-inferiority of overall survival of laparoscopic distal gastrectomy on locally AGC has been confirmed by many clinical trials (40–42), few data are available for laparoscopy total gastrectomy (LTG) for AGC and even fewer for laparoscopic surgery for AGC with metastasis. The Chinese Laparoscopic Gastrointestinal Surgery Research Group (CLASS) conducted a randomized controlled trial (LASS-01) of laparoscopic gastrectomy for stage T2–T4a gastric cancer with or without lymph node metastasis or distant metastasis in China and compared treatment with laparoscopy and open distal gastrectomy (43). The results indicated that laparoscopy was safe and effective (43). However, there are no relevant systematic studies examining the feasibility of laparoscopic surgery alone in progressive gastric cancer with metastasis. Our patient underwent chemo-immune combination therapy, and it is still controversial whether to perform Laparoscopic surgery in these patients due to the lack of systematic clinical trials. But we have tried several cases in our clinical practice with good results. The final dissemination will require a formal Phase III clinical study to confirm its effectiveness.

This is the first case of AGC with supraclavicular metastasis that achieved CR after treatment with tislelizumab combined with XELOX. Tislelizumab was approved by the National Medicinal Products Administration (NMPA) on December 2019 for the treatment of Hodgkin’s lymphoma in China. By blocking PD-L1/PD-L2-associated cell signaling to avert immune responses, tislelizumab promotes the production of cytokine and restores the clearing ability of T-cells, thus causing tumor cell death (44). Immunotherapy with tislelizumab has been well-established in the treatment of malignant melanoma, lung cancer, and clear-cell kidney cancer, but its role in the treatment of esophageal and gastric cancer is uncertain. Our patient with metastatic GC achieved CR with much better outcomes than most clinical studies of metastatic GC, based on especially CR rate (9). The patient in this study responded positively to this combination therapy and continues to demonstrate disease control, and the additional survival time reflects the superiority of the treatment scheme in this case.

## Conclusion

Whether tislelizumab has bright prospects in the immunotherapy of gastric cancer and whether the watch-and-wait (WW) method can be applied to treatment of patients with AGC who achieved complete clinical remission (CCR) remain controversial. However, some cases have demonstrated their efficacy (24, 35). In conclusion, we reported a 63-year-old retired female of AGC with supraclavicular metastasis that successfully achieved CR through chemotherapy and immunotherapy, indicating the potential molecular biomarkers and providing possibilities of applying the watch-and-wait (WW) method to patients who are older or in poor physical condition. Nonetheless, supported only by several cases, our conclusions should be proved by accumulations of more cases with similar regimens as well as comparisons with the case characteristics of the successful use of tislelizumab to achieve CR through which the conclusions may better contribute to precision treatment of GC.

## Data availability statement

The original contributions presented in the study are included in the article/Supplementary Material. Further inquiries can be directed to the corresponding authors.

## Ethics statement

Written informed consent was obtained from the individual(s) for the publication of any potentially identifiable images or data included in this article.

## Author contributions

ZZ researched data and wrote the manuscript; ZZ, P-LD, and ZL analyzed the inspection data and contributed to the discussion; ZL, SH, and P-LD reviewed and modified the manuscript; YW prepared **Figures 1**–**3**. All authors reviewed the manuscript. All authors contributed to the article and approved the submitted version.
